# Age decreases mitochondrial motility and increases mitochondrial size in vascular smooth muscle

**DOI:** 10.1113/JP271942

**Published:** 2016-04-09

**Authors:** Susan Chalmers, Christopher D. Saunter, John M. Girkin, John G. McCarron

**Affiliations:** ^1^ Strathclyde Institute of Pharmacy & Biomedical SciencesUniversity of StrathclydeGlasgowG4 ONRUK; ^2^ Department of PhysicsDurham UniversitySouth RoadDurhamDH1 3LEUK

## Abstract

**Key points:**

- Age is proposed to be associated with altered structure and function of mitochondria; however, in fully‐differentiated cells, determining the structure of more than a few mitochondria at a time is challenging. In the present study, the structures of the entire mitochondrial complements of cells were resolved from a pixel‐by‐pixel covariance analysis of fluctuations in potentiometric fluorophore intensity during ‘flickers’ of mitochondrial membrane potential.
- Mitochondria are larger in vascular myocytes from aged rats compared to those in younger adult rats.
- A subpopulation of mitochondria in myocytes from aged, but not younger, animals is highly‐elongated.
- Some mitochondria in myocytes from younger, but not aged, animals are highly‐motile.
- Mitochondria that are motile are located more peripherally in the cell than non‐motile mitochondria.

**Abstract:**

Mitochondrial function, motility and architecture are each central to cell function. Age‐associated mitochondrial dysfunction may contribute to vascular disease. However, mitochondrial changes in ageing remain ill‐defined because of the challenges of imaging in native cells. We determined the structure of mitochondria in live native cells, demarcating boundaries of individual organelles by inducing stochastic ‘flickers’ of membrane potential, recorded as fluctuations in potentiometric fluorophore intensity (flicker‐assisted localization microscopy; FaLM). In freshly‐isolated myocytes from rat cerebral resistance arteries, FaLM showed a range of mitochondrial X‐Y areas in both young adult (3 months; 0.05–6.58 μm^2^) and aged rats (18 months; 0.05–13.4 μm^2^). In cells from young animals, most mitochondria were small (mode area 0.051 μm^2^) compared to aged animals (0.710 μm^2^). Cells from older animals contained a subpopulation of highly‐elongated mitochondria (5.3% were >2 μm long, 4.2% had a length:width ratio >3) that was rare in younger animals (0.15% of mitochondria >2 μm long, 0.4% had length:width ratio >3). The extent of mitochondrial motility also varied. 1/811 mitochondria observed moved slightly (∼0.5 μm) in myocytes from older animals, whereas, in the younger animals, directed and Brownian‐like motility occurred regularly (215 of 1135 mitochondria moved within 10 min, up to distance of 12 μm). Mitochondria positioned closer to the cell periphery showed a greater tendency to move. In conclusion, cerebral vascular myocytes from young rats contained small, motile mitochondria. In aged rats, mitochondria were larger, immobile and could be highly‐elongated. These age‐associated alterations in mitochondrial behaviour may contribute to alterations in cell signalling, energy supply or the onset of proliferation.

## Key points

- Age is proposed to be associated with altered structure and function of mitochondria; however, in fully‐differentiated cells, determining the structure of more than a few mitochondria at a time is challenging. In the present study, the structures of the entire mitochondrial complements of cells were resolved from a pixel‐by‐pixel covariance analysis of fluctuations in potentiometric fluorophore intensity during ‘flickers’ of mitochondrial membrane potential.
- Mitochondria are larger in vascular myocytes from aged rats compared to those in younger adult rats.
- A subpopulation of mitochondria in myocytes from aged, but not younger, animals is highly‐elongated.
- Some mitochondria in myocytes from younger, but not aged, animals are highly‐motile.
- Mitochondria that are motile are located more peripherally in the cell than non‐motile mitochondria.

**Abbreviations**
FaLM: flicker‐assisted localization microscopy
MBE: minimum bounding ellipse
TMRE: tetramethyl‐rhodamine ethyl ester

## Introduction

Vascular function declines with age as a result of a range of morphological and molecular alterations, such as vessel wall intima‐media thickening, smooth muscle cell hyperplasia, endothelial cell dysfunction, increased oxidative stress and decreased nitric oxide bioavailability. These factors combine to contribute to both acute and chronic cardiovascular diseases, the risk of which sharply increases with age. Age‐associated alterations in vascular smooth muscle cells include altered contractility, extra‐cellular matrix synthesis, apoptosis and senescence, combined with a switch to a more inflammatory, proliferative and migratory phenotype (Lacolley *et al*. 2012). Stiffening of the cells themselves (Sehgel *et al*. 2015) may determine vessel stiffness, particularly in the microcirculation where there is low extracellular matrix (Anghelescu *et al*. 2015). Vascular smooth muscle cells also have an increased production of oxidative species with age, both via the activity of plasma membrane NADPH oxidase and the mitochondria; indeed, mitochondrial superoxide production may precede, and then induce, NADPH superoxide production (Mistry *et al*. 2013). Attenuating this mitochondrial oxidant production inhibits vascular smooth muscle cell switching to a proliferative and migratory phenotype and, conversely, reducing mitochondrial anti‐oxidants promotes proliferation (Wang *et al*. 2012). Another early alteration in smooth muscle phenotypic switching is a change in the intracellular dynamics of mitochondria (Chalmers *et al*. 2012; Marsboom *et al*. 2012) to a more highly‐motile state. The molecular mechanics of mitochondrial motility have been well characterized: the organelles attach to the microtubule‐associated, ATP‐hydrolysing motor proteins kinesin or dynein via various combinations of adaptor proteins (Miro, Milton/TRAK, syntabulin) that dissociate upon elevation of cytosolic calcium concentration (Lin & Sheng, 2015). The extent of mitochondrial motility in fully‐differentiated, post‐mitotic cells, however, is less clear (Chalmers *et al*. 2012; Song & Dorn, 2015) and it remains to be seen whether alterations in mitochondrial motility are associated with vascular smooth muscle ageing.

Mitochondria also have the ability to undergo fusion and fission events (Kasahara & Scorrano, 2014), which both alter the morphology of individual mitochondria and are proposed to maintain the health of the mitochondrial population via inter‐mixing of mitochondrial DNA and the subsequent targeting of any dysfunctional daughter mitochondria for mitophagy (Mishra & Chan, 2014). Mitochondria accumulate damage to their DNA, proteins and lipid components that can eventually limit function, particularly in long‐lived cells. It is not clear whether such damage results in mitochondrial structural changes or motility alterations, each of which could alter interactions with other cellular components or functions, such as the ability to locally provide ATP, to modify calcium buffering or shuttle ions and metabolites.

Assessing mitochondrial structure and motility in fully‐differentiated cells is challenging. We developed methods to measure mitochondrial shape and motility in native, freshly‐isolated vascular smooth muscle cells by exploiting the electrical signatures of each organelle to restore structure (Chalmers *et al*. 2015). Transient depolarizations of mitochondrial membrane potential (‘flickers’) arise from transient opening of the mitochondrial permeability transition pore (Duchen *et al*. 1998; O'Reilly *et al*. 2004). These flickers are stochastic electrical events and can be recorded optically using rapidly‐partitioning potentiometric fluorophores such as tetramethyl‐rhodamine ethyl ester (TMRE). Using flickers, the structure of individual mitochondria was resolved in a manner similar to previous work with cultured endothelial cells (Gerencser & Adam‐Vizi, 2005) but with additional functionalities that enabled the visualization of the high number of small mitochondria present in native smooth muscle cells. Individual mitochondria could be discriminated even when images of multiple mitochondrial structures overlapped because only regions that were electrically‐contiguous displayed fluorescence changes with matching temporal profiles.

In the present study, we used image‐processing routines to extract spatial information about mitochondrial structure, implemented via the Python programming language (https://www.python.org). Pixels were selected manually as putative organelle centres and then the temporal changes in TMRE fluorescence intensity of each centre pixel were compared with the temporal changes in the 1599 pixels surrounding that putative centre. The covariance of the time series was used as the comparison metric. Boundaries of individual, electrically‐discrete mitochondria were defined as contiguous regions of covariance exceeding a threshold of +0.4, resolving individual organelles within otherwise visually‐unclear clusters. Although very different in implementation, this technique was inspired by the super‐resolution imaging techniques of photo‐activation localization microscopy (PaLM) and blink‐assisted localization microscopy (BaLM) (Betzig *et al*. 2006; Burnette *et al*. 2011) and is hence referred to as flicker‐assisted localization microscopy (FaLM). In the present study, we have applied this technique to compare mitochondrial structure in vascular smooth muscle cells from young and aged adult rats. To explore possible mitochondrial shape changes, an additional analysis routine was created to automatically quantify the extent of elongation of each organelle. Mitochondria were larger and more elongated in aged animals. Mitochondrial dynamics were also examined. Unexpectedly, differences in mitochondrial motility were also observed: there was a decreased extent of movement in aged *vs*. young animals. The increased mitochondrial size may partly explain the decrease in motility, as may changes to mitochondrial positioning: those mitochondria that were motile were more commonly found in the cell periphery than were non‐motile mitochondria. These changes in mitochondrial dynamics and structure may contribute to the changes in vascular performance with age.

## Methods

### Ethical approval

We understand the ethical principles under which *The Journal of Physiology* operates and our work complies with the animal ethics policy and checklist as outlined recently (Grundy, 2015). All experiments were carried out on tissue collected immediately after death (as described below) of animals not subject to any other treatments. Animal maintenance and killing were in accordance with UK regulations [Animals (Scientific Procedures) Act 1986, revised under European Directive 2010/63/EU; death in accordance with ASPA Schedule 1 in the UK and in Annex IV in the European Directive] and, as such, were fully compliant with ethical requirements.

### Animals

Ten male Sprague–Dawley rats (five at 3 months of age and five at 18 months of age; from an in‐house colony allowed *ad libitum* access to food and water) were killed by trained technicians with an i.p. overdose of sodium pentobarbital (Euthatal, 200 mg kg^−1^; Merial, Lyon, France; Schedule 1 listed method of dispatch) prior to rapid removal of the brain into Mops‐buffered saline [145 mm NaCl, 2 mm 3‐(*N*‐morpholino)propanesulfonic acid), 1.2 mm NaH_2_PO_4_, 4.7 mm KCl, 1.17 mm MgCl_2_, 5 mm glucose, 0.02 mm EDTA and 2 mm CaCl_2_, pH 7.4].

### Cell isolation

The superior cerebellar and posterior cerebral arteries from one animal were combined and smooth muscle cells enzymatically dispersed by incubation in isolation buffer (55 mm NaCl, 80 mm Na‐glutamate, 6 mm KCl, 1 mm MgCl_2_, 10 mm glucose, 10 mm Hepes, 0.2 mm EDTA and 0.1 mm CaCl_2_, 1.24 mg ml^−1^ BSA, pH 7.3, 34.5°C) containing 2.2 mg ml^−1^ type F collagenase and 1 mg ml^−1^ hyaluronidase for 14 min, followed by a second incubation in isolation buffer containing 1.7 mg ml^−1^ papain and 0.7 mg ml^−1^ dithioerythritol for 14 min. Tissue was washed three times in isolation buffer and then three times in the same without BSA. Individual myoctes were released by gentle trituration, stored at 4°C and used within 6 h or, alternatively, were allowed to grow in standard cell culture conditions (Chalmers *et al*. 2012). There was one enzymatic isolation carried out per animal.

### Fluorescence imaging

Cells were loaded with TMRE (62.5 nm) in a chambered #1 coverglass (Lab‐Tek; Thermo Fisher Scientific Inc., Waltham, MA, USA) in isolation buffer (20 min, 37°C) and then imaged on an inverted epifluorescence microscope (TE2000‐U; Nikon, Tokyo, Japan) with a 100× 1.3 NA S‐Fluor oil objective. Only cells with a smooth, relaxed morphology were included in the present study (routinely 50–75% of cells released during the isolation procedure). The output of a xenon arc lamp monochromator system (555 ± 5 nm; PTI Inc., Birmingham, NJ, USA), guided via an optical light guide through a field stop diaphragm before being reflected off a long‐pass dichroic mirror (Chroma Inc., Bellows Falls, VT, USA) and reflective from 550–570 nm and transmissive >580 nm, illuminated the field. Emitted light was imaged by a Photometrics Cascade 512B CCD camera (actual pixel size 16 μm, effective pixel size 225 nm at the sample; Roper Scientific, Planegg, Germany) controlled by WinFluor, version 3.4.5 software (J. Dempster, University of Strathclyde, Glasgow, UK). Images were acquired at 10 Hz. All imaging was carried out at 37°C in a heated environmental chamber plus objective heater (Tokai Hit, Fujinomiya, Japan).

### Image processing

**Figure 1:**
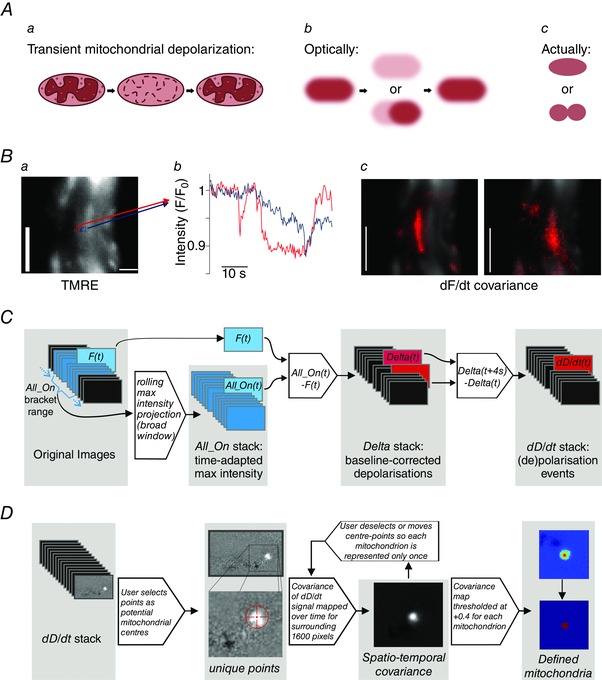
**Resolving individual mitochondria using flickers of mitochondrial membrane potential, FaLM** *A*, transient ‘flickers’ of the mitochondrial membrane potential, such as during transient mitochondrial permeability transition pore opening (*a*), and as visualized with a membrane‐potential sensitive fluorophore (*b*), enable discrimination of discrete, tightly‐packed mitochondria (*c*). *B*, mitochondria within a freshly‐isolated vascular smooth muscle cell loaded with TMRE (62.5 nm, *a*) display localized fluctuations in fluorescence intensity (*b*, measured in the neighbouring 3 × 3 pixel regions shown in *a*) that result in discrete spatio‐temporal covariance maps around the two points (*c*); scale bars = 5 μm. *C*, the image processing workflow to extract mitochondrial de‐ and re‐polarization events involves the creation of a rolling wide time window maximum‐intensity projection stack (*All_On*), against which each frame of the original stack (*F*_t_) is compared to create the *Delta* stack of baseline‐corrected fluorescence changes; this *Delta* stack is then differentiated over a 4 s time window to reveal de‐ and re‐polarization events (d*D*/d*t*). *D*, in FaLM, the user guides the selection of mitochondrial centres, with an examination of d*D*/d*t* signals in a 40 × 40‐pixel region around the potential individual mitochondrion. Each spatially separated pixel within the region is considered to image the same mitochondrion if (1) the covariance of its temporal d*D*/d*t* signal with the d*D*/d*t* signal of the chosen organelle centre, as defined by the centre of the region, is above some threshold and (2) the pixel under examination is connected to the centre of the region by other such pixels. A threshold of +0.4 was employed.

Individual mitochondria were identified and visualized even from optically‐cluttered images of overlapping organelles by utilizing ‘flickers’: transient depolarizations of the mitochondrial membrane potential (Fig. 1 *A*), recorded as localized intensity fluctuations of the rapidly‐repartitioning fluorophore TMRE (Fig. 1 *B*). For FaLM (Chalmers *et al*. 2015), multiple stages of image processing were carried out using custom‐written algorithms in Python. First, to counteract minor X‐Y cell movement or dye bleaching, an *All_On* image stack was created using a broad‐time‐window rolling maximum intensity projection. Each frame of the original image stack was then compared with its time‐referenced point in the *All_On* stack to create an image stack (the *Delta* image stack) of deviations from fully‐polarized mitochondria. The onset and end of flickers were then revealed by differentiating the *Delta* stack over a 4 s window (Fig. 1 *C*). Peaks and troughs in the resultant d*D*/d*t* stack were used to identify individual mitochondria (Fig. 1 *D*). The temporal d*D*/d*t* signal in an area larger than the organelle (40 × 40 pixel area around a central point in each mitochondrion) was used to determine the boundary of each mitochondrion by making a spatial map of the covariance of the d*D*/d*t* from each pixel with the d*D*/d*t* from the central pixel. From this covariance map, the size, shape and position could be determined. The boundary threshold was set at a covariance of +0.4.

Equations and numerical and algorithmic basis underpinning FaLM:

1) The input time series of images are defined as a 3D array ***I*(*x*, *y*, *t*),** where (*x*, *y*) are integer pixels’ co‐ordinates defining the spatial domain of *nx* by *ny* pixels (0 < = *x* < *nx*, 0 <= *y* < *ny*) and *t* is the integer frame index corresponding to the time domain covering *n* frames (0 < = *t* < *n*).
2) An array is then calculated, ***All_On*(*x*, *y*, *t*)**, over the same spatial and temporal range. This array estimates the signal that would be seen at a given pixel (*x*, *y*) and time (*t*) if all organelles were emitting fluorescence. This is achieved by making an averaged measurement of that pixel's maximum intensity over some time window. The pseudo‐code for calculating *All_On* is given below and is performed for every value of *x*, *y* and *t* to generate a 3D signal array.

define ***All_On*(*x*, *y*, *t*)**:

***t*0** = ***t* – window_size** and ***t*1** = ***t* + window_size** *# Find the start and end of the search window, window_size is in units of frames, typically equal to 60 s*
**if *t*0 < 0 then *t*0** = **0** *# Limit the search window to the start of the data*
**if *t*1 >** = ***n* then *t*1** = ***n* – 1** *# Limit the search window to the end of the data*
*# Extract the image signal for pixel (x, y) between t0 and t1*
**signal** = ***I*[*x*, *y*, *t*0:*t*1]** *# Values from each timepoint between t0 and t1*
**signal** = **arithmetic mean of brightest 20% of values in signal**
**return signal**

3) The array Delta(*x*, *y*, *t*) gives a measure of instantaneous depolarization and is defined as **Delta(*x*, *y*, *t*)** = ***All_On*(*x*, *y*, *t*) – *I*(*x*, *y*, *t*)**.

4) The time derivative of the Delta array, d*D*/d*t*(*x*, *y*, *t*) gives an indication of the rate of change of depolarization, and is defined as:

$$\begin{array}{ccc} dD/dt\left(x,y,t\right) & = & \mathrm{Delta}\left(x,y,t+dt/2\right)\\ & & -\mathrm{Delta}(x,y,t-dt/2) \end{array}$$

where d*t* is the number of frames corresponding to the time period used for differentiation (we used 4 s). Care is taken to choose a time period such that d*t*/2 is an integer.

5) Given a manually chosen seed point (*xs*, *ys*) a covariance map COVMAP(*x*, *y*) is generated over a region of space of 40 × 40 pixels centred on that point (*xs* − 20 < = *x* < *xs* + 20, *ys* − 20 < = *y* < = *ys* + 20):

$$\begin{array}{ccc} \mathrm{COVMAP}\left(x,y\right) & = & \mathrm{COVARIANCE}\left(dD/dt\left(x,y\right),\right.\\ & & \left.dD/dt\left(\mathrm{xs},\mathrm{ys}\right)\right) \end{array}$$

where COVARIANCE(*a*, *b*) is the covariance of two signals *a* and *b*, and d*D*/d*t*(*x*, *y*) refers to the time series of the d*D*/d*t* array at the spatial co‐ordinates (*x*, *y*) over all values of *t*.

6) The covariance map is then normalized to a scale of ±1 by dividing it by its maximum value. This discards the information on the variance of the mitochondrion's polarization and information on its peak intensity; these two values are inseparably mixed. The resultant array COVMAP_NORM represents the relative intensity map of the mitochondrion:

$$\begin{array}{ccc} \mathrm{COVMAP}\_\mathrm{NORM}\left(x,y\right) & = & \mathrm{COVMAP}\left(x,y\right)\\ & & /\max\left(\mathrm{COVMAP}\right) \end{array}$$

7) Finally, all pixels are compared to a threshold of +0.4, with the resulting binary mask forming an image of the mitochondrion's extent.
8) To exclude the occasional distant pixel that may exceed the set threshold (given the noisy nature of the data), a flood fill algorithm that confined the binary pixel mask to only those forming a contiguous area with the central pixel, with connections recognized on any of the remaining eight surrounding pixels.

A source code release is in preparation and will be freely available via the University of Strathclyde data repository ‘KnowledgeBase’ (https://pure.strath.ac.uk/portal/en/datasets). In the meantime, researchers are encouraged to contact the authors if they wish to share the current tools described above.

Cell area was determined by comparing the area in pixels of a cell mask and a mitochondrial complement mask. For each cell, a mask was created by tracing the outline of a brightfield image of the cell. A mitochondrial complement mask was created by taking the area from every individual mitochondrial mask created by the FaLM tracing procedure.

To analyse mitochondrial shape change with age, the minimum bounding ellipse (MBE) was then identified for each binary pixel mask using the GroopM Python module (Imelfort *et al*. 2014). The MBE is the smallest ellipse that contains all points above the binary threshold, with the centre of the ellipse, the length of the minor and major axes and the axis rotation being free parameters.

Mitochondrial motility was measured by manually examining the image stacks and counting the number of mitochondria observed to move (independent of any whole‐cell movement) over a 10 min period, as a percentage of the total number of mitochondria observed in the cell from FaLM analysis. Distance of mitochondria within the cell was measured by evaluating a signed distance function from the cell edge, as determined by manual tracing in MetaMorph, version 7.5.0.0 (Molecular Devices, Sunnyvale, CA, USA), using the fast marching method implemented in the ‘scikit‐fmm’ Python module.

### Statistical analysis

The results are presented as the mean ± SEM (or SD, noted in text). On some occasions, the mode (i.e. the most frequently occurring value) was also provided where this was most informative, for *n* mitochondria within each group, with the number of cells and number of animals (and hence cell isolations) also provided. Statistical differences of mean mitochondrial areas were determined by a two‐sample Kolmogorov–Smirnov test and considered significant at *P* < 0.05. For all other mean values, differences were determined by a paired Students *t* test and considered significant at *P* < 0.05. Statistical differences of proportions were determined by an unpaired two‐sample *t* test and considered significant at *P* < 0.05. Tests were carried out in OriginPro, version 9 (OriginLab Corp., Northampton, MA, USA).

## Results

### Mitochondrial architecture

**Figure 2:**
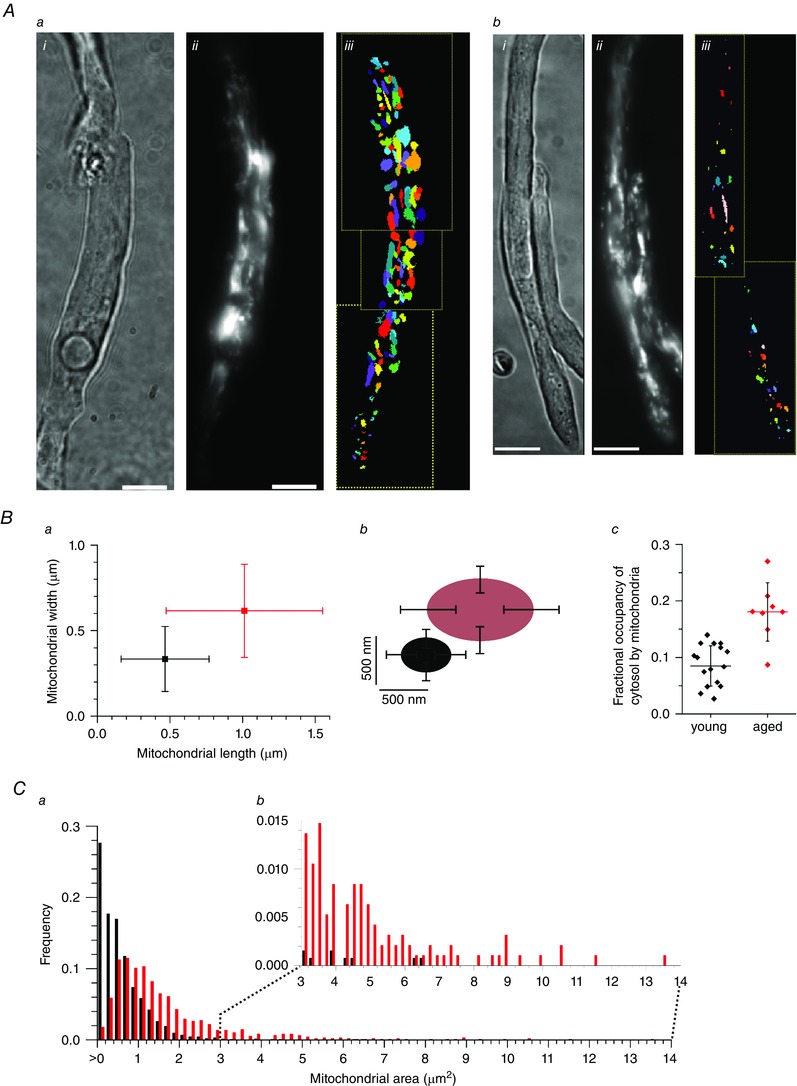
**Mitochondrial size increases with age** *A*, mitochondria in single, freshly‐isolated smooth muscle cells from cerebral resistance arteries from aged rats (18 months; *a*) were larger than those in younger animals (3 months, *b*); *i*, brightfield; *ii*, TMRE; *iii*, FaLM‐defined mitochondrial shapes. The organelles have been colour coded for display purposes to permit neighbouring mitochondria to be distinguished. Scale bars = 10 μm. *B*, mitochondria in cells from aged rats were longer, wider and occupied a greater proportion of the cell than in young animals; length and width represented graphically (*a*) and pictorially (*b*); fractional occupancy of the cell by mitochondria (*c*) was measured as the cell area/total mitochondrial area defined by FaLM (horizontal bar and whiskers are mean ± SD). *C*, the frequency distribution of mitochondrial area (*a*) shows that the majority of mitochondria in cells from younger rats (black) were very small but, in cells from aged rats (red), the population of mitochondria shifted to larger sizes, with noticeably more very large mitochondria (*b*).

Mitochondrial architecture was examined in vascular smooth muscle cells from young adult and aged adult rats (3 and 18 months, respectively) using FaLM. Native vascular smooth muscle cells freshly‐isolated from cerebral resistance arteries were loaded with TMRE (62.5 nm, below the self‐quench limit of ∼100 nm in these cells), imaged and then the mitochondrial architecture determined as described in the Methods and as shown in Fig. 1. Resolving the structure of mitochondria from the conventional fluorescence image (Fig. 1) was problematic. FaLM resolved the structure of the entire cell's mitochondrial complement. Each spatially separated pixel within the region was considered to image the same mitochondrion if (1) the covariance of its temporal d*D*/d*t* signal with the d*D*/d*t* signal of the chosen organelle centre, as defined by the centre of the region, was above some threshold and (2) the pixel under examination was connected to the centre of the region by other such pixels. A threshold of +0.4 was employed. Mitochondria from young animals were dispersed throughout the cell (Fig. 2 *Aa*), largely presenting as spheres or short rods. The geometric mean area of the organelles in young animals was 0.35 ± 0.017 μm^2^. Individual mitochondria were significantly larger in cells from aged animals compared to younger animals (mean area of 1.17 ± 0.05 μm^2^, significantly different compared to young, *P* < 0.001, *Z*‐value = 11.13; *n* ≥ 950 mitochondria from ≥10 cells and five animals each) (Fig. 2). In cells from aged animals, there were fewer very small mitochondria and more particularly large organelles (Fig. 2 *C*). The mode mitochondrial area in cells from young animals was 0.051 μm^2^ compared to 0.710 μm^2^ in cells from aged animals. Ninety‐five percent of mitochondria in cells from young animals had an area <1.65 μm^2^ compared to <4.75 μm^2^ in cells from aged animals. The largest 5% of mitochondria in cells from young animals had a mean area of 2.37 ± 0.11 μm^2^ compared to 6.69 ± 0.3 μm^2^ in cells from aged animals (significantly different, *P* < 0.001, *Z*‐value = 5.18; *n* = 70 mitochondria from young and 48 from aged animals). The density of mitochondria within the two cell populations was estimated on a cell‐by‐cell basis as the whole‐cell area/total area of mitochondria in that cell and was significantly higher in cells from aged animals (mean ± SD fraction of the cell occupied by mitochondria was 0.181 ± 0.052, *n* = 9 in aged animals compared to 0.085 ± 0.032, *n* = 15, in cells from young animals, *P* < 0.001).

**Figure 3:**
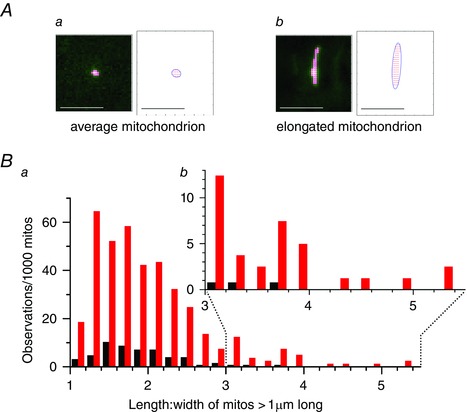
**Some mitochondria are highly‐elongated in aged animals** *A*, mitochondrial length and width measurements obtained automatically in a Python routine that fitted a minimum bounding ellipse to the set of all pixels for each mitochondrion defined by FaLM. An example average‐sized mitochondrion (*a*) is shown in comparison with an example elongated mitochondrion from a myocyte from an aged rat (*b*); non‐mitochondrial pixels masked in dark green, the pixel corners within a mitochondrion are marked as red dots and the entire organelle is bounded within an ellipse drawn as a dark blue line. Scale bars = 5 μm. *B*, 39.5% of the mitochondria in myocytes from aged rats (red, *a*) were >1 μm in length, compared to 5.4% in younger rats (black). Inset (*b*) shows the increased number of highly‐elongated mitochondria (length:width > 3) in aged animals.

On closer inspection of the data, a subpopulation of mitochondria that were particularly highly‐elongated in cells from aged animals became apparent: 5.3% of mitochondria were >2μm long in aged animals compared to 0.15% in young animals (proportions significantly different, *P* < 0.001, *Z*‐value = 7.98; *n* ≥ 950 mitochondria from ≥10 cells and five animals for each). To quantify the extent of mitochondrial elongation, the length and width of each mitochondrion was measured (custom Python script described in Methods). Figure 3 *A* shows example mitochondria and dimension measurements of average‐sized mitochondria and a highly‐elongated mitochondrion from an aged animal. Summarized ellipticity measurements of young and aged cell mitochondria are shown in Fig. 3 *B*. In aged animals, 4.2% of mitochondria in smooth muscle cells had a length:width ratio >3 compared to only 0.4% of mitochondria in cells from young animals (proportions significantly different, *P* < 0.001, *Z*‐value = 5.21; *n* ≥ 950 mitochondria from ≥10 cells and five animals for each).

### Mitochondrial motility

**Figure 4:**
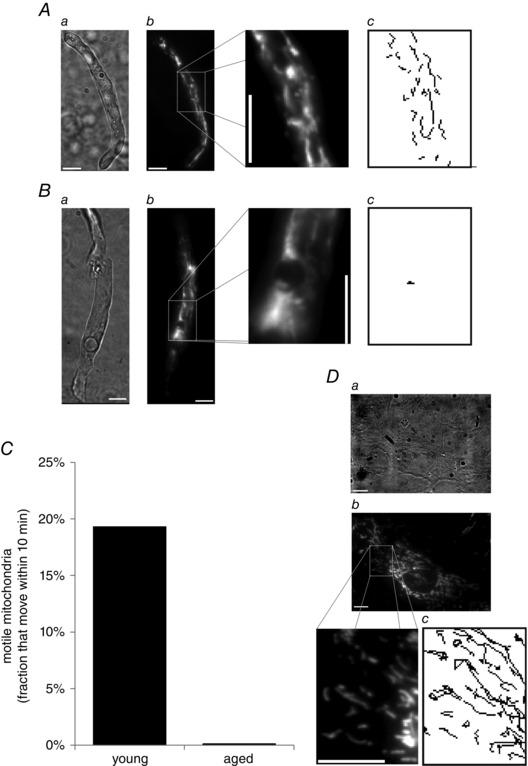
**Mitochondria in native vascular smooth muscle cells from young rats are often motile, whereas those from aged animals only occasionally display limited motion** *A*, in freshly‐isolated smooth muscle cells (brightfield, *a*) from cerebral resistance arteries from 3‐month‐old rats, mitochondria (TMRE, 62.5 nm, *b*) were highly‐motile; mitochondrial motion tracks recorded over a 5 min period are shown in (*c*). *B*, in freshly‐isolated smooth muscle cells from 18‐month‐old rats, however, mitochondrial motion was observed infrequently; the example shown in (*c*) is the only mitochondrion that was motile in the entire cell (*a*, brightfield; *b*, TMRE; *c*, motion tracks over 5 min period). *C*, summarized fraction of motile mitochondria per 10 min period. *D*, as a further comparison, mitochondria in cultured vascular smooth muscle cells are very highly motile (*a*, brightfield; *b*, TMRE; *c*, motion tracks over a 5 min period at the same scale as *A* and *B*). Scale bars = 10 μm.

When investigating mitochondrial structure using FaLM, we observed that a significant fraction of the mitochondria in the vascular smooth muscle cells from younger animals was motile. An example of the motion tracks derived from a 5 min recording period is shown in Fig. 4 *A* and in the Supporting information (Movie S1). The motility took the form of random motion interspersed with bursts of directed motion. In cells from young animals, directed mitochondrial motion at speeds up to 95 nm s^−1^ and up to 12 μm distance in a single burst was observed, although the majority of motion observed was <1.5 μm and commonly appeared to comprise Brownian‐like random motion. This motility was only observed during the initial recordings for each cell that were made with very low excitation light intensity (0.7 nW μm^–2^). Subsequently, a neutral density filter was removed from the excitation lightpath (intensity increased to 6.7 nW μm^–2^) and mitochondrial motility halted. These observations suggest that light may inhibit mitochondrial movement. In comparison, under the same experimental conditions (0.7 nW μm^–2^), mitochondria in cells from aged animals were rarely motile; indeed, the sole motile mitochondrion out of 811 examined is shown in Fig. 4 *B* and and in the Supporting information (Movie S1). This mitochondrion moved over a much shorter distance (∼500 nm) than the organelles in cells from young animals, preventing accurate measurements of velocity. Summarized data in Fig. 4 *C* show that 19% of mitochondria moved within 10 min in cells from young animals (*n* = 1135 mitochondria, 17 cells, five animals) compared to 0.12% of mitochondria in cells from aged animals over the same time period (*n* = 811 mitochondria, seven cells, five animals, significantly different compared to young animals, *P* < 0.001, *Z*‐value = 13.2). By contrast, vascular smooth muscle cells maintained in proliferative cell culture conditions were highly‐motile regardless of light intensity (Fig. 4 *D*; see also the Supporting information, Movie S1), with almost all mitochondria moving within a 10 min period. We have previously shown mitochondrial speeds in the order of 1000 nm s^−1^ with bursts of directed motion that can displace mitochondria up to 5000 nm (Chalmers *et al*. 2012).

**Figure 5:**
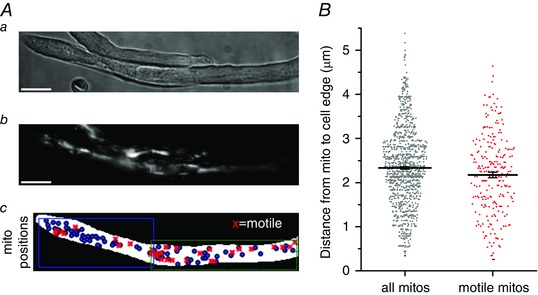
**Motile mitochondria are located more peripherally than the average mitochondrion** *A*, native vascular smooth muscle cells (brightfield, *a*) from young adult rats contain mitochondria (TMRE, *b*), a fraction of which were observed to move (*c*, motile mitochondria, red crosses; all mitochondria identified by FaLM, blue circles) within the cell. *B*, summarized data shows the range of mitochondrion‐to‐cell edge distances for all mitochondria identified by FaLM (grey circles) and for those mitochondria that were motile (red crosses).

We finally examined whether there was any relationship between the intracellular positioning of motile *vs*. non‐motile mitochondria in cells from the younger rats. The proximity of each mitochondrion to the nearest cell edge was measured and correlated with motility as described in the Methods. The mean distance of mitochondria from the plasma membrane was 2.17 ± 0.06 μm for the motile mitochondria compared to 2.33 ± 0.03 μm for all mitochondria (*n* = 220 motile mitochondria, 1069 total mitochondria, from 17 cells, five animals; mean values significantly different, *P* = 0.02) (Fig. 5). These results show that mitochondria that are motile in cells from younger animals are positioned more peripherally than mitochondria that are not motile.

## Discussion

Vascular dysfunction accompanies age and is a risk factor for the development of cardiovascular disease. There is increased arterial stiffness and media thickness with age, which are features attributed to a progressive increase in vascular smooth muscle hypertrophy and collagen content (Cliff, 1970). Although the changes in vascular structure are acknowledged, mechanisms underlying the changes in vascular performance are not fully understood.

Mitochondria play important roles in biosynthetic pathways, cellular energetics, cellular redox and Ca^2+^ signalling, as well as the regulation of programmed cell death. Mitochondria are particularly susceptible to damage over time (Lopez‐Lluch *et al*. 2008) and age‐related changes in mitochondria probably alter several cellular physiological functions. The structure of mitochondria is central to both the organelles and cell function, and mitochondria exist in a variety of arrangements (Rizzuto *et al*. 1998; De Giorgi *et al*. 2000; Guillery *et al*. 2008; Peng *et al*. 2011; McCarron *et al*. 2013). For example, the rapid formation of fragmented mitochondria is induced early in apoptosis (Frank *et al*. 2001; Karbowski & Youle, 2003), whereas giant or highly interconnected mitochondria have also been observed in human liver diseases such as cirrhosis (Tandler & Hoppel, 1986). However, little is known about how age may affect mitochondrial structure in vascular smooth muscle. Therefore, we tested the hypothesis that advancing age may alter mitochondrial structure in vascular resistance arteries in young *vs*. old rats. To resolve mitochondrial structure, we used ‘flickers’ of membrane potential, recorded as fluctuations in cationic fluorophore intensity (FaLM), to measure the organelles shape and position from their electrical signature. FaLM provides a convenient and rapid method for determining the structure of the entire mitochondrial complement of a cell.

Significant changes in the structure of mitochondria occur with age in the present study. In smooth muscle cells from young animals, mitochondria were mostly very small compared to the organelles' size in aged animals. In older animals, the smooth muscle cells also contained a subpopulation of highly‐elongated mitochondria (>2 μm long). Elongated mitochondria were seen only rarely in younger animals. The mitochondria occupied 18% of the cell area in cells from aged animals, whereas, in cells from younger animals, the organelles only occupied 8.5% of the cell. Our results are consistent with previous findings in which larger and giant mitochondria were observed in aged rat adrenal cortical cells (Murakoshi *et al*. 1985).

The size and shape of mitochondria is determined by the processes of fusion and fission. Fission and fusion permit morphological transitions from small individual structures to short rods through to interconnected complex tubular networks and vice versa (Peng *et al*. 2011; McCarron *et al*. 2013). Fission is regulated by proteins such as hFis1 and Drp1, as well as fusion, mitofusin 1 and 2 (MFN1 and 2) and OPA1 (Hoppins *et al*. 2007). Despite significant research efforts identifying the proteins involved in mitochondrial fission and fusion, the precise relationship between mitochondrial shape and cellular function is only partly understood and a wealth of apparently contradictory views exist (McCarron *et al*. 2013). Nonetheless, mutations in genes controlling mitochondrial fusion are associated with human disease. Mutations in Mfn2 were found in patients with Charcot‐Marie‐Tooth neuropathy type 2A (Zuchner *et al*. 2004; Kijima *et al*. 2005) and mutations in the *OPA1* gene cause dominant optic atrophy (Alexander *et al*. 2000; Delettre *et al*. 2000). These findings suggest that there is a link between mitochondria length and cell function.

Changes in mitochondrial length occur in cell models of ageing. Cell cultures enter a state of cellular ‘senescence’ after a finite number of divisions. In the senescence state, cells are growth arrested and display several biochemical and morphological changes suggestive of ageing, and the cell senescence state is viewed as a model of ageing. Increased mitochondrial length occurs in the process of senescence and experimentally increasing mitochondrial length induces a senescence‐like state. For example, in Chang and HeLa cell cultures, depletion of hFis1 by RNA interference resulted in elongation of mitochondria and the development of a sensescence‐associated cell phenotype (Lee *et al*. 2007). There was also a decreased mitochondrial membrane potential, increased generation of reactive oxygen species and DNA damage (Lee *et al*. 2007). Reversing the increased length of mitochondria in hFis1 knockdown cells, by inhibition of mitochondrial fusion (silencing genes encoding OPA1), triggered extensive mitochondria fragmentation in Chang cells and significantly reduced the senescent cell phenotype (Lee *et al*. 2007). Elongated giant mitochondria have been also observed in senescence models induced by treatment with desferoxamine or hydrogen peroxide and are correlated with a reduction in hFis1 levels during senescent progression (Yoon *et al*. 2006). Interestingly, when Chang cells were exposed to prolonged low level subcytotoxic concentration of reactive oxygen species, mitochondrial length increased and the cells gained senescent phenotypes (Yoon *et al*. 2006). With age, vascular smooth muscle cells also have an increased production of reactive oxygen species as a result of the activity of plasma membrane NADPH oxidase and mitochondria (Mistry *et al*. 2013). Perhaps the increased reactive oxygen species may lead to increased mitochondrial length. The changes in mitochondrial length in smooth muscle cells from aged animals may occur because of an enhanced fusion process or reduced fission, or both. One proposal for the physiological significance of the increased mitochondria length in aged tissues is that fusion continuously suppresses the contribution of defective mitochondria, hence maintaining cell function (Ono *et al*. 2001). Alternatively, the increased mitochondrial length in age may provide protection against apoptosis (Frank *et al*. 2001; Brooks *et al*. 2009; Karbowski, 2010). FaLM provides a snap‐shot of mitochondrial architecture at a particular point in time; however, rearrangements of the organelles may occur (e.g. at mitochondrial contact points; Picard *et al*. 2015) that have the potential to be identified by multiple rounds of FaLM imaging.

In addition to increasing size, the extent of mitochondrial motility decreased with age in the present study. A similar observation has been made in neurons (Gilley *et al*. 2012). In smooth muscle cells in young animals, mitochondrial motility occurred as random motion Brownian motion interspersed with bursts of directed motion. Directed and Brownian‐like mitochondrial motility occurred regularly in cells from young animals, with ∼20% of the organelles moving during the 10 min period recording period. On the other hand, only 0.1% of mitochondria in smooth muscle from older animals moved in the recording period. In cultured cells, almost all of the mitochondria moved. The question arises as to why mitochondria appear to move more in cells from young than old animals. In a previous study, we found relatively little movement of mitochondria in non‐proliferative smooth muscle cells and extensive movement for cells in a proliferative state (Chalmers *et al*. 2012). The observation led us to suggest that motion was a requirement for proliferation to occur (Chalmers *et al*. 2012). On the basis of the present findings, it is tempting to speculate that, as animals age, it will be more difficult for smooth muscle cells to re‐enter the cell cycle for vascular repair.

Motor‐driven displacement of mitochondria has been linked to positioning the organelle at locations where there is an energy demand (Hollenbeck & Saxton, 2005; MacAskill & Kittler, 2009). The positioning of mitochondria may also regulate Ca^2+^ signalling in smooth muscle cells (Chalmers & McCarron, 2008; Olson *et al*. 2010). Interestingly, the extent of mitochondrial movement in smooth muscle cells from young animals was greatest in those organelles that were located near the periphery of cells. Intriguing recent research has shown that stem cells may spatially‐segregate their mitochondria by organelle age, with more young mitochondria being located at the peripheral aspects of cells (Katajisto *et al*. 2015). Hence, motility may be linked to the age of the individual organelles themselves (Katajisto *et al*. 2015). Interestingly, in this respect, mitochondrial biogenesis itself is impaired in aged smooth muscle cells (Ungvari *et al*. 2008), which would tend to shift the populations towards ‘older’ organelles. Light, in the presence of the fluorophores used to visualize mitochondria, appeared to inhibit the organelles movement in young animals but not in cultured cells in the present study. The reason for the susceptibility of organelle movement in young animals is not clear. Light, together with the fluorophore, will produce reactive oxygen species. Perhaps movement is more sensitive to reactive oxygen species in native cells from young animals compared to cultured cells.

The data reported in the present study show significant alterations in mitochondrial structure and dynamics in vascular smooth muscle as animals age. Although the present study does not provide the mechanism, changes in mitochondrial dynamics and structure may arise from factors that include both the direct and indirect effects of ageing, such as progressive impairment of mitochondrial function, immune activation, increased oxidative stress, altered Ca^2+^ concentrations or the accumulation of advanced glycation end products (Tosato *et al*. 2007). Whatever the mechanism, the changes in mitochondrial structure and dynamics may then alter cell signalling, energy supply or proliferation, and impede re‐entry into the cell cycle, contributing to the decrease in vascular performance with age.

## Additional information

### Competing interests

The authors declare that they have no competing interests.

### Author contributions

SC, CDS, JMG and JGM conceived and designed the experiments. SC and CDS collected, assembled, analysed and interpreted the data. SC, CDS, JMG and JGM drafted, critically revised and approved the final version of the manuscript. Experiments were performed in the laboratory of JGM. All authors agree to be accountable for all aspects of the work, ensuring that questions related to the accuracy or integrity of any part are appropriately investigated and resolved. All persons designated as authors qualify for authorship, and all those who qualify for authorship are listed.

### Funding

This research was supported by The Wellcome Trust (092292/Z/10/Z) and British Heart Foundation (PG/11/70/29086 and a Research Excellence Award to Durham/Edinburgh University).

## Supporting information


**Movie S1. Mitochondria in native vascular smooth muscle cells from young rats are commonly motile, whereas those from aged animals only occasionally display limited motion**
Left: in freshly‐isolated smooth muscle cells from cerebral resistance arteries from 3‐month‐old rats, mitochondria (TMRE, 62.5 nm) regularly displayed intracellular motion. Middle: in the same cells from 18‐month‐old rats, however, only the occasional motile mitochondrion was observed; this example shows the only mitochondrion that was motile in the entire cell (just below mitochondrial‐free nuclear region). Right: as a further comparison, mitochondria are extraordinarily motile in cultured vascular smooth muscle cells. For all sections, y‐axis field of view = 20 μm.
